# HER2 as a potential therapeutic target on quiescent prostate cancer cells

**DOI:** 10.1016/j.tranon.2023.101642

**Published:** 2023-02-18

**Authors:** Kenji Yumoto, Jibraan Rashid, Kristina G. Ibrahim, Steven P. Zielske, Yu Wang, Maiko Omi, Ann M. Decker, Younghun Jung, Dan Sun, Henriette A. Remmer, Yuji Mishina, Laura A. Buttitta, Russell S. Taichman, Frank C. Cackowski

**Affiliations:** a Department of Periodontics and Oral Medicine, University of Michigan School of Dentistry, Ann Arbor, MI 48109, USA; b Michigan State University College of Human Medicine, East Lansing, MI 48824, USA; c Department of Biological and Materials Sciences, University of Michigan School of Dentistry, Ann Arbor, MI 48109, USA; d Department of Internal Medicine, Division of Hematology and Oncology, University of Michigan School of Medicine, Ann Arbor, MI 48109, USA; e Department of Molecular, Cellular and Developmental Biology, University of Michigan, Ann Arbor, MI, 48109, USA; f Proteomics & Peptide Synthesis Core, Biomedical Research Core Facilities, University of Michigan Medical School, Ann Arbor, MI 48109, USA; g Department of Periodontics, University of Alabama at Birmingham School of Dentistry, Birmingham, AL 35233, USA; h Wayne State University School of Medicine and Karmanos Cancer Institute Department of Oncology, Detroit, MI 48201, USA

**Keywords:** Dormancy, HER2, ERBB2, Quiescent, Prostate cancer, Disseminated tumor cells, Cell cycle, G0

## Abstract

Quiescent prostate cancer (PCa) cells are common in tumors but are often resistant to chemotherapy. Quiescent PCa cells are also enriched for a stem-like tumor initiating population, and can lead to recurrence after dormancy. Unfortunately, quiescent PCa cells are difficult to identify and / or target with treatment in part because the relevant markers are intracellular and regulated by protein stability. We addressed this problem by utilizing PCa cells expressing fluorescent markers for CDKN1B (p27) and CDT1, which can separate viable PCa cells into G_0_, G_1_, or combined S/G_2_/M populations. We used FACS to collect G_1_ and G_0_ PC3 PCa cells, isolated membrane proteins, and analyzed protein abundance in G_0_ vs G_1_ cells by gas chromatography mass spectrometry. Enrichment analysis identified nucleocytoplasmic transport as the most significantly different pathway. To identify cell surface proteins potentially identifying quiescent PCa cells for future patient samples or for antibody based therapeutic research, we focused on differentially abundant plasma membrane proteins, and identified ERBB2 (HER2) as a cell surface protein enriched on G_0_ PCa cells. High HER2 on the cell membrane is associated with quiescence in PCa cells and likely induced by the bone microenvironment. Using a drug conjugated anti-HER2 antibody (trastuzumab emtansine) in a mouse PCa xenograft model delayed metastatic tumor growth, suggesting approaches that target HER2-high cells may be beneficial in treating PCa. We propose that HER2 is deserving of further study in PCa as a target on quiescent cells to prevent recurrence, decrease chemotherapy resistance, or eradicate minimal residual disease.

## Highlights

• Quiescent cells largely contribute to tumor progression, metastasis, and relapse.
• Known quiescence markers are intracellular, not viable for antibody targeting.
• Plasma membrane protein HER2 is upregulated in quiescent cells.
• Drug conjugated anti-HER2 antibody delayed metastasis formation in mice.

## Introduction

Despite continued incremental progress, prostate cancer (PCa) remains a significant public health concern, as it causes approximately 34,500 deaths per year in the United States alone [1]. While cancer is generally thought of as a disease of cellular overproliferation, significant levels of cellular heterogeneity exist in tumors, and this includes the presence of many non-proliferative cells. In particular for PCa, many cells in the tumor may be in transient states of G_0_ where they enter and exit quiescence in response to microenvironmental cues. For example, in a series of 512 primary PCa tumors, 82% of tumors had more than 95% of their cells negative for the proliferative marker Ki67 [2]. Quiescent cells have important roles for progression of PCa and other malignancies. These include proposed roles in maintenance of disseminated PCa cells and potential metastatic recurrence after curative intent prostatectomy. Although not possible to measure directly, at least some patient bone marrow isolated prostate cancer disseminated cells appear to be quiescent from bioinformatics analyses [3]. Horning et al found that patients with a quiescence-associated gene signature derived from single cell RNA sequencing paradoxically had a shorter time to recurrence in the TCGA prostate adenocarcinoma dataset. They also found that the quiescence-associated cell population had stem-like characteristics and was less sensitive to inhibition of the androgen receptor [4]. Similarly, quiescent or slowly cycling prostate cancer cells in mouse bone marrow identified by label retention are more resistant to immunotherapy [5]. Lastly, at least in the case of breast cancer cell lines, quiescent cells are also more resistant to cytotoxic chemotherapy in addition to having some stem-like characterisctics [6], [7], [8].

However, quiescent PCa cells remain difficult to identify, especially in live cultures or tumors. The cell cycle drivers; cyclins, cyclin dependent kinases, and their Cip/Kip inhibitors are intracellular and regulated predominantly by phosphorylation or protein stability and therefore are not amenable to analysis by live cell flow cytometry or interrogation of single cell RNA sequencing datasets. Similarly, the canonical marker of proliferative cells, Ki67, also has an intracellular location and is therefore also not accessible for antibody based live cell flow cytometry. In addition to the difficulty in identifying quiescent PCa cells for basic research, the intracellular location of cell cycle regulatory proteins prevents them from being used to selectively target quiescent cells by antibodies, bispecific antibodies, antibody drug conjugates, chimeric antigen receptor T cells (CAR-T), and the radio-ligand classes of therapeutics. All of these therapeutic classes are increasingly utilized, but require accessibility of a targeting protein on the plasma membrane outer leaflet [9], [10], [11].

A major step forward in the study of cell cycle and especially G_0_ arose with the development of fluorescent reporters for cell cycle regulatory proteins, which permits live cell imaging throughout the cell cycle and isolation of individual cell cycle populations by FACS, without need for cell cycle synchronization by serum starvation. A particularly useful combination of markers for the study of G_0_ is the combination of a peptide fragment of the DNA replication licensing protein CDT1 conjugated to mCherry that is targeted for degradation during S, G2 and M-phases, and a mutated version of Cip/Kip CDKN1B (p27) that lacks cell cycle inhibitor activity conjugated to mVenus developed by Oki et al, which is targeted for degradation during G1 phase [12]. In this reporter system, dual positive cells are in G_0_, CDT1-mCherry singly positive cells are in G_1_ and early S and dual negative cells are in either S, G_2_, or M phases . We made use of this marker system in PCa using the PC3 cell line and observed that G_0_ PC3 cells are enriched for PCa stem cell markers, and that known regulators of PCa dormancy affect G_0_ entry [13]. We commonly term these as “PC3 Venus-Cherry” cells.

In the current study, we set out to better understand quiescent PCa cells in G_0_, and ideally discover a cell surface marker for quiescent PCa cells that could serve as a therapeutic target. To accomplish this, we used FACS to separate PC3 Venus-Cherry cells into G_0_ and G_1_ populations and isolated membrane-bound proteins. We chose to selectively study G_1_ vs. G_0_ because in many assays, the G_0_ and G_1_ populations are indistinguishable and the Venus-Cherry reporter system allows us to separate the G_0_ population. We performed gas chromatography (GC) followed by mass spectrometry (MS) based proteomics on the isolated proteins and described the overall results using gene ontology and pathway analyses of the differentially expressed genes. Because purification of membrane proteins also includes intracellular membrane bound proteins, we then concentrated our analyses on proteins predicted to be in the plasma membrane outer leaflet and expressed more highly in G_0_ than G_1_ cells. Surprisingly, one of these proteins is ERBB2 (Her2), which we chose to further evaluate due to the wealth of clinical and investigational drugs available for this target. We found that Her2 is expressed more highly in quiescent cells than cycling cells for several PCa cell lines and that an anti-Her2 antibody drug conjugate significantly delayed tumor formation after cardiac left ventricle injection of PC3 cells into SCID mice as a xenograft model.

## Results

**Fig. 1:**
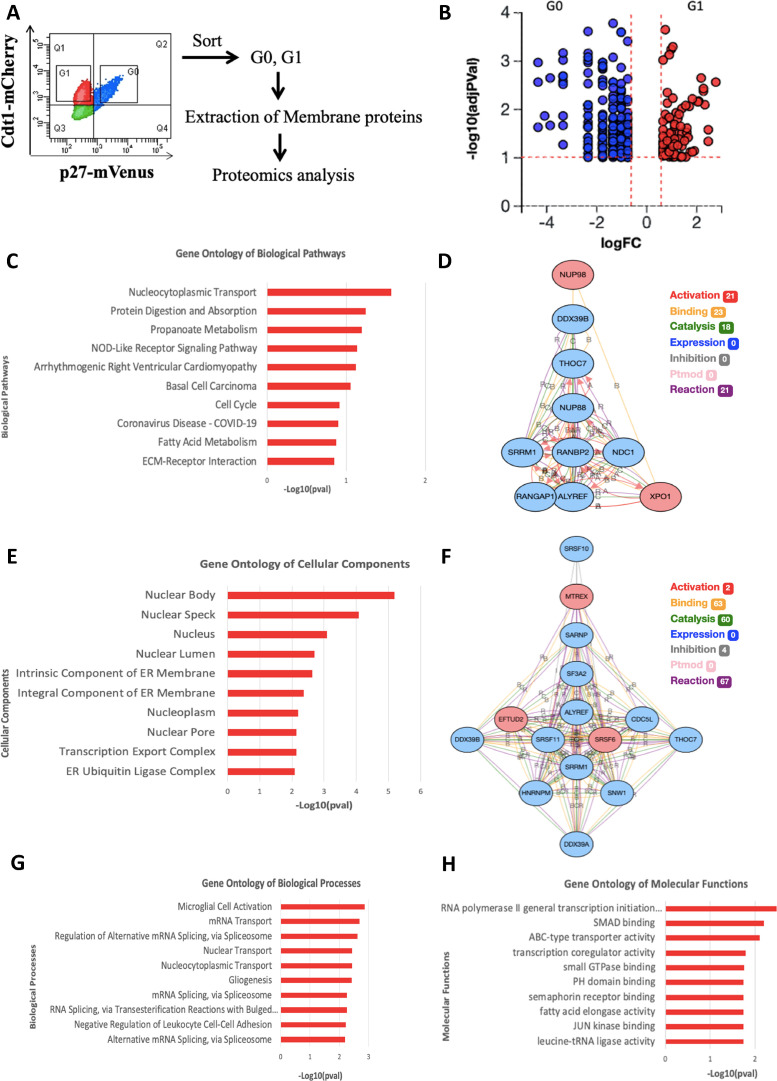
Proteomics of membrane proteins in G_0_ vs. G_1_ PC3 cells. (A) Experimental schema. (B) Volcano plot of 354 differentially expressed (DE) genes plotted as measured expression log_10_ fold change against statistical significance of the change. All genes had non-zero expression detected in G_0_ and G_1_, fold change ≥4 or ≤ 0.25 and *p* ≤ 0.1 Fisher's exact test. Data is from three pooled biologic replicates. (C) Gene ontology analyses of DE genes using KEGG database, shown are the ten biological pathways most significantly impacted by DE genes. (D) Theoretical network analyses for nucleocytoplasmic transport—the most significantly enriched pathway from panel (C). (E) Gene ontology analyses of DE genes using the gene ontology cellular components database. (F) Theoretical network analyses for nuclear body—the most significantly associated cellular component from panel (E). For the network analyses in (D) and (F), modes of theoretical interaction and numbers of interaction are shown in the legend. On the network analysis web, nodes with increased expression in G_0_ are blue and increased expression in G_1_ are red. (G) Gene ontology analyses of DE genes using gene ontology biological processes database. (H) Gene ontology analyses of DE genes using the molecular functions database.

*GC-MS Proteomic analysis of membrane proteins from G_0_ vs. G_1_ PCa cells*. To discover potential new cell surface markers and therapeutic targets in quiescent PCa cells, we used PC3 cells stably transduced and clonally selected carrying fluorescent reporters for Cip/Kip CDKN1B (p27) conjugated to mVenus and a peptide from DNA replication licensing factor CDT1 conjugated to mCherry, which we term “PC3 Venus-Cherry” cells and have recently described in detail [13]. In biological triplicate experiments, we cultured PC3 Venus-Cherry cells in the presence of 5% fetal bovine serum (FBS) for 48 hours, and isolated the G_1_ CDT1-mCherry singly positive population and the G_0_ p27-mVenus CDT1-mCherry dual positive populations by fluorescence activated cell sorting (FACS). We then isolated total membrane proteins and pooled the triplicate samples to increase peptide yield. The G_0_ vs. G_1_ populations were analyzed by gas chromatography mass spectrometry (GC mass spec / GC-MS) (Fig. 1A). Initially, 3,791 proteins were identified (Supplemental table 1). To summarize the results of the proteomics data, we analyzed differentially expressed (DE) proteins for enrichment in gene ontology and signaling pathway groups. We imposed analysis thresholds of a four-fold change in levels, *p* < 0.1 and non-zero expression in both G_0_ and G_1_, with 354 proteins meeting these criteria (Fig. 1B). Of the Kyoto Encyclopedia of Genes and Genomes (KEGG) pathway groups, the DE proteins were most significantly enriched in the “nucleocytoplasmic transport” group (Fig. 1C), suggesting unexpected changes may occur in nucleocytoplasmic transport between G_0_ and G_1_. Consistent with this, there are known, conserved roles for misregulation of Nucleoporins such as Nup88, Nup98, RanBP2 and its partners such as Xpo1 in tumorigenesis - as previously reviewed [14], [15], [16]. For other commonly studied gene ontology groups: “nuclear body” was the most significantly enriched for cellular components (Fig. 1E), “microglial cell activation” was the most enriched for biological processes (Fig. 1G), and “RNA polymerase II general transcription initiation factor binding” was most enriched for molecular functions (Fig. 1H). For the predicted biological interactions amongst the DE proteins enriched in each gene ontology group, RANBP2 was the central node for biological pathways (Fig. 1D) and ALYREF, SRSF11, SRSF6, and SRRM1 were central nodes for cellular components (Fig. 1F).

*Proteomics data was sorted and HER2 was selected for further exploration.* The proteomics data is based on a preparation of total membrane-associated proteins – including proteins bound to intracellular membranes. Thus, with our goal in mind of finding novel cell surface targets for quiescent PCa cell identification and treatment, we manually sorted the proteomics data to focus on predicted plasma membrane proteins. We selected predicted plasma membrane proteins that were at least four-fold differentially expressed, or had zero reads in one sample and had a *p* value for differential expression of < 0.1. This included 40 proteins that were higher in G_0_ cells (Table 1) and 28 proteins that were higher in G_1_ cells (Table 2). We noticed ERBB2 (HER2) among the plasma membrane proteins with increased expression in G_0_ and decided to further study this protein because of its known importance in other malignancies, especially breast cancer [17], [18], [19], [20] and because of the many drugs available that target it [18,21].

**Fig. 2:**
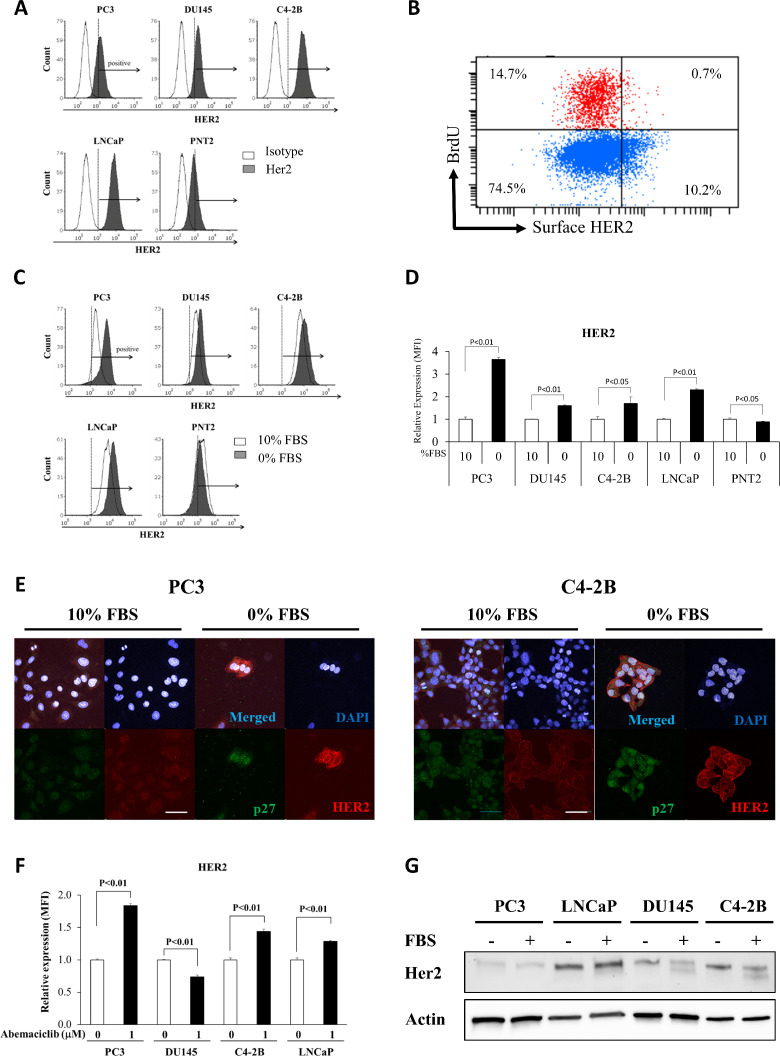
Cell surface HER2 expression on quiescent prostate cancer cells. (A) Cell surface HER2 expression on PCa cell lines (PC3, DU145, C4-2B, LNCaP) and benign prostate epithelial cells (PNT2) cultured in 10% FBS measured by flow cytometry and mean fluorescence intensity (MFI). Open histograms represent an isotype control antibody. Shaded histograms are a HER2 antibody. The vertical line and arrow represent positive expression as defined by the isotype control antibody. (B) Flow cytometry for surface HER2 and BrdU incorporated into DNA of PC3 cells (C) Histograms showing HER2 cell surface expression on the same cell lines cultured in the presence or absence of FBS. (D) Quantification of HER2 surface expression from panel (B) as defined by mean fluorescence intensity (MFI). (E) Immunofluorescence imaging of PC3 (left) or C4-2B (right) cells cultured in either 10% FBS or serum starved conditions showing endogenous p27 and HER2 expression. HER2 (red), p27 (green) and nuclei (DAPI, blue). Scale bar, 50 μm. (F) Cell surface HER2 as measured by flow cytometry of PCa cells cultured with either 1 µM abemaciclib or 0.01% ethanol vehicle. (G) Western blot for HER2 from indicated cell lines cultured for 2 days with or without FBS. β-actin was a loading control. Data represent mean ± S.D. of triplicate wells. A representative biological replicate experiment is shown.

*Cell surface HER2 is increased by independent methods of quiescence induction and is correlated with p27 levels.* Using flow cytometry, we found that HER2 was expressed on the cell surface of all PCa cell lines analyzed: PC3, DU145, C4-2B, and LNCaP, as well as the benign prostate epithelial cell line, PNT2 – visualized by higher labeling as compared to an isotype control antibody (Fig. 2A). To further validate that Her2 is increased on the cell surface of the quiescent cells within a population, we used BrdU to identify cells that had synthesized DNA and performed flow cytometry for BrdU and Her2. To specifically examine cell surface Her2, cells were labeled with the Her2 antibody prior to fixation and permeabilization (which was required to detect BrdU incorporated into DNA). As expected from the proteomics data, a lower proportion of the BrdU positive cells were positive for Her2 than the BrdU negative cells (Fig. 2B). We then determined if PCa cell surface HER2 expression was induced by cellular quiescence by two independent methods. First, we induced quiescence by three days of serum starvation vs. culture in full serum. Cell surface HER2 expression was increased in the PCa cell lines (PC3, DU145, C4-2B and LNCaP) but not PNT2 benign prostate epithelial cells upon serum starvation (Fig. 2C and D). Similarly using immunofluorescence, we also observed that HER2 levels were increased and primarily localized to the cell surface by culture in serum free conditions in both PC3 and C4-2B cells (Fig. 2E). Because we were interested in quiescence rather than cell death, we verified that serum starvation for 3 days reduced cell number but did not induce cell death in PCa cells (Supplemental Fig. 1A and 1B). Serum starvation also increased expression of endogenous p27, a cell cycle inhibitor and molecular marker of G_0_ cells, and target of the fluorescent reporter system used in the proteomics experiment (Supplemental Fig. 1C and 1D and Fig. 2E). To determine if PCa HER2 surface expression is increased by a different method of quiescence induction, we used abemaciclib - an FDA-approved drug for the treatment of advanced or metastatic breast cancers, which selectively inhibits the cyclin dependent kinases CDK4/6 and promotes entry into G_0_ [22,23]. Overall, cell surface HER2 was significantly increased when cells were treated by abemaciclib, with the exception of the DU145 cell line (Fig. 2F). As expected, treatment of PCa cells with abemaciclib significantly reduced cell numbers (Supplemental Figure 1E). For both LNCaP and C4-2B, 20-30% of the cells had died during the treatment period, but PC3 and DU145 were seemingly resistant at the concentrations used (Supplemental Fig. 1F). To determine if the total amount of HER2 (as opposed to cell surface only) we performed Western blots for HER2 of PC3, LNCaP, DU145, and C4-2B cells grown for two days in the presence or absence of serum and observed similar levels of total HER2 with serum starvation (Fig. 2G). Therefore, we conclude that cell surface HER2, but not necessarily total HER2, expression is increased by independent methods of G_0_ induction.

**Fig. 3:**
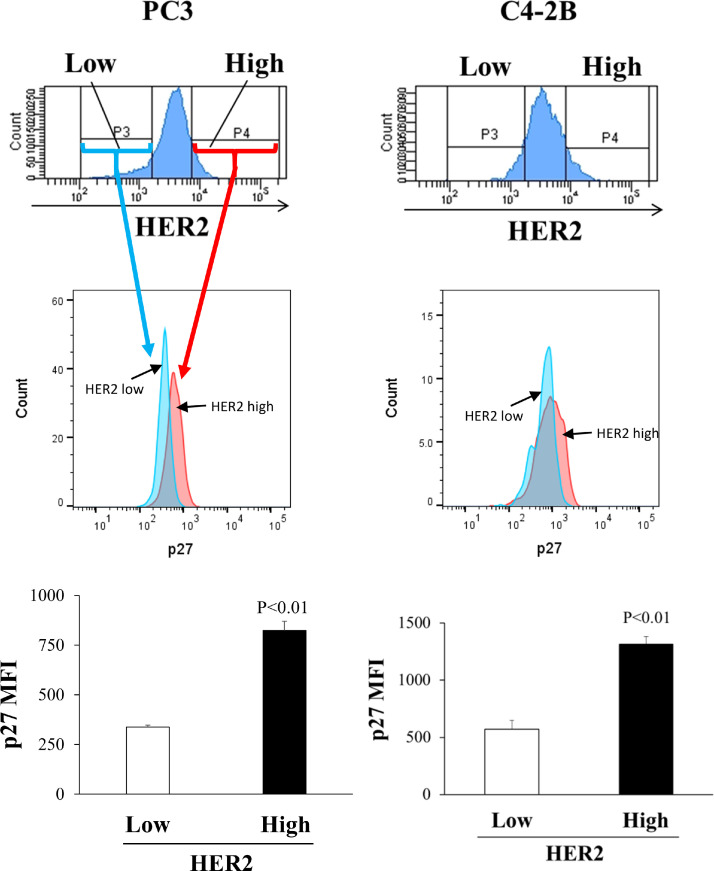
Cell surface HER2 expression is associated with p27 expression. Left: PC3, Right: C4-2B. Top: Flow cytometry histograms demonstrating cell surface (labeling before fixation) HER2 expression on PC3 and C4-2B cells. Cells were gated by lowest 10% or highest 10% of HER2 levels (HER2 low and HER2 high) populations. Middle: Example flow cytometry histograms of p27 labeling from either the lowest (blue) or highest (red) 10% of HER2 surface expression. Bottom: Quantified p27 expression in the low vs. higher HER2 populationsp27 levels were measured as mean fluorescence intensity (MFI). Data represent mean ± S.D. of triplicate wells. A representative biological replicate experiment is shown.

*HER2 cell surface expression is associated with the expression of p27*. To further validate our finding that cell surface HER2 is increased in quiescent PCa cells, we examined correlation of cell surface HER2 with endogenous p27. The original proteomics experiment utilized a p27 mutant fluorescent reporter. However, here we examine endogenous p27 in fixed and permeabilized cells. We in part validated the specificity of the p27 antibody for fixed and permeabilized cells by showing higher labeling as compared to an isotype control (Fig. S1 C) and showing that p27 labeling increased with serum starvation (Fig. S1 D). We labeled cell surface HER2 and intracellular p27 with fluorescent antibodies on PC3 and C4-2B cells cultured in serum free conditions and analyzed with flow cytometry. HER2 was first labeled in viable cells and p27 was then labeled after fixation and permeabilization. For both cell lines, p27 fluorescence intensity was much higher in cells with the top 10% of surface HER2 as compared to the cells with the lowest 10% of HER2 (Fig. 3). Additionally, in agreement with the flow cytometry results, strong expression of HER2 was detected by immunofluorescence imaging on the surface of both p27 positive PC3 and C4-2B cells, whereas in the cells cultured in 10% FBS, expression of p27 and HER2 was reduced (Fig. 2E).

**Fig. 4:**
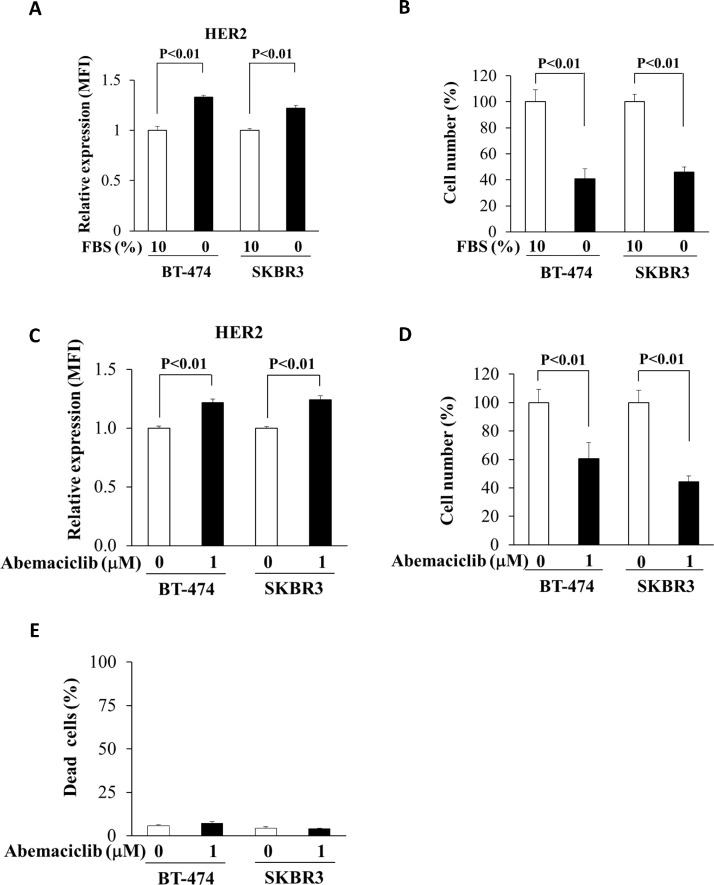
Breast cancer quiescence induction and cell surface HER2 expression. (A) Mean fluorescence intensity (MFI) for HER2 as measured by flow cytometry in breast cancer cell lines after 3 days of culture in full serum or serum free conditions. (B) Viable cell count by trypan blue exclusion for the experiment in panel (A). (C) HER2 cell surface expression in breast cancer cells treated with or without abemaciclib. (D) and (E) Viable cell count and % dead cells by trypan blue exclusion in the experiment from panel (C). Data represent mean ± S.D. of triplicate wells. A representative biologic replicate is shown.

*Induction of quiescence also increases HER2 in breast cancer cell lines*. Although we focused this work on PCa we also questioned if some of these findings might also extend to breast cancer, where HER2 has been more widely studied and targeted. Therefore, we examined whether serum starvation and abemaciclib treatment increased cell surface HER2 levels in the HER2^+^ breast cancer cell lines BT-474 and SKBR3. As seen in PCa cells, serum starvation increased HER2 cell surface expression in breast cancer cell lines (Fig. 4A-B). Likewise, cell cycle inhibition also increased cell surface HER2 in breast cancer cell lines and in this case, did not significantly increase cell death (Fig. 4C-E). Therefore, our findings might also be useful in breast cancer and other malignancies.

**Fig. 5:**
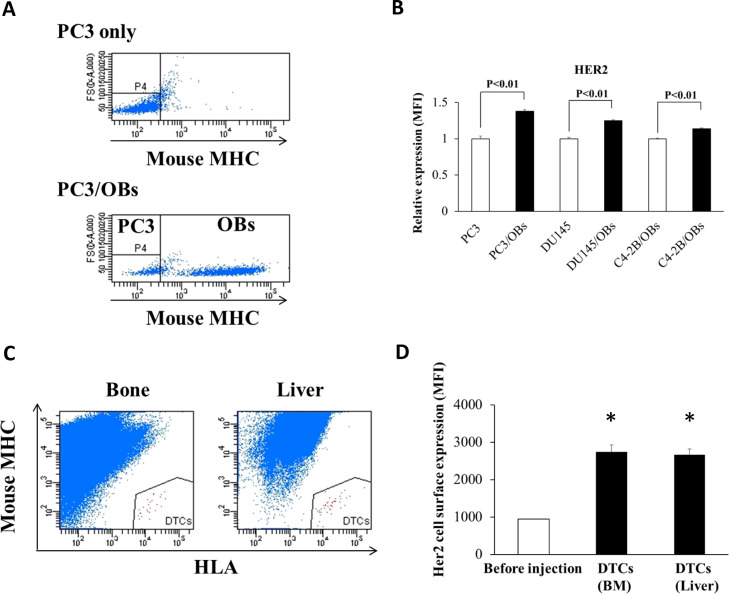
Microenvironmental induction of cell surface HER2. (A) Human PC3 PCa cells were cultured with murine OBs, MC3T3-E1 cells for 3 days in the presence of 10% FBS, and PCa cells were identified as murine MHC negative populations. (B) Relative HER2 expression (MFI) on PC3, DU145, or C4-2B PCa cells co-cultured with OBs, compared to PCa cells cultured alone. Data represent mean ± S.D. of triplicate wells. A representative biological replicate experiment is shown. (C) DTCs were identified in BM and liver using FACS as human HLA positive and mouse MHC negative populations. (D) Cell surface HER2 expression on DTCs isolated from BM and liver. Data are shown as mean ± S.D. (N=3). * indicates *p* < 0.05 compared to control cells.

*Cell surface HER2 is induced in the metastatic prostate cancer microenvironment*. PCa commonly disseminates to bone and can remain dormant in the bone before eventual recurrence. We have previously shown that signals from the bone marrow microenvironment can promote G_0_ and quiescence in PCa [13]. We therefore wanted to examine whether the bone marrow microenvironment may increase levels of HER2 on the cell surface in PCa. Osteoblasts are critical components of the bone marrow niche and thought to play a significant role in regulating disseminated tumor cell (DTC) proliferation in the marrow [24,25]. Previously we demonstrated that a murine pre-osteoblastic cell line, MC3T3-E1, dramatically decreases the proliferation of PCa cells when cultured together and induces G_0_ [26]. Therefore, we analyzed HER2 cell surface expression of PCa cells when cultured with MC3T3-E1. To allow separate analysis of the two cell types, the murine MC3T3-E1 cells were stained by an antibody against the strain specific haplotype of the mouse major histocompatibility complex, and cell surface HER2 levels were assayed on the MHC-negative human PCa cells. Co-culture with MC3T3-E1 cells increased cell surface HER2 on PC3, DU145, and C4-2B cells as compared to cells cultured without MC3T3-E1 cells (Fig. 5A-B). To examine cell surface expression of HER2 on DTCs directly, PC3 cells were injected into male CB.17. SCID mice by left ventricle intracardiac (i.c.) injection. After 48 hours, the pelvis, femora, tibiae and liver were collected. Cells were obtained from the bones and liver and stained using human leukocyte antigen (HLA) antibodies and mouse MHC antibodies. PCa cells were identified as human HLA positive, and mouse-MHC negative cells (Fig. 5C), and the cell surface expression of HER2 on DTCs was measured by flow cytometry. Compared to PC3 cells before injection, HER2 cell surface levels were significantly increased on PCa cells derived from mouse bone marrow and liver (Fig. 5D).

**Fig. 6:**
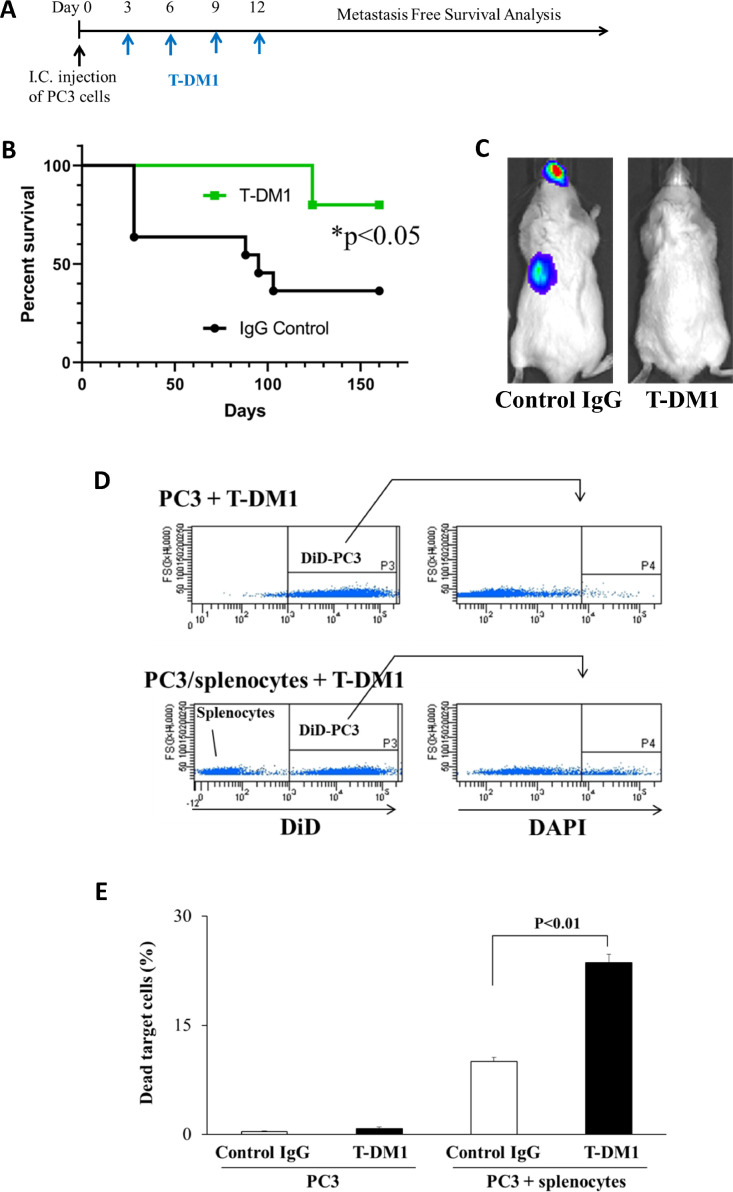
Impact of T-DM1 treatment on metastasis free survival in a prostate cancer left ventricle injection xenograft model. (A) Experimental design for the *in vivo* experiment. GFP/luciferase-expressing PC3 cells were injected into male SCID mice by left ventricle intracardiac (i.c.) injection. Human IgG (15 mg/kg) or T-DM1 (15 mg/kg) were injected by intraperitoneal (i.p.) injection every 3 days until 12 days after tumor injection IgG: *n* = 12. T-DM1: *n* = 14. (B) Kaplan-Meier analysis of time to formation of metastases visible by bioluminescence imaging or death. (C) Representative bioluminescence images of control mice (human IgG injected) and T-DM1 treated mice 124 days after tumor injection. (D) Antibody dependent cellular cytotoxicity (ADCC) assays using flow cytometry. PC3 cells were labeled with DiD for subsequent identification, and were treated with T-DM1 before addition of splenocytes from SCID mice. PC3 cells were identified as DiD positive and non-viable cells were identified as positive for DAPI. (E) Dead PC3 cells (%) quantified from data in panel (D) Data represent mean ± S.D. (N=3).

*Targeting HER2 reduces metastasis in an animal model.* To determine if disseminated quiescent PCa cells expressing HER2 might be a useful therapeutic target, we evaluated the impact of the HER2 targeted antibody drug conjugate trastuzumab emtansine (T-DM1) on a PCa mouse model of metastasis. In this model, luciferase-labeled PC3 cells were injected in the left ventricle of SCID mice, and the mice were treated with T-DM1 alone or IgG control up to day 12 after tumor inoculation (Fig. 6A). Mice treated with control IgG began to demonstrate the first evidence of metastasis by 4 weeks, and only 40 % of the mice survived without metastasis after 160 days (Fig. 6B). By contrast, T-DM1 treatments significantly inhibited the development of metastasis with 80% of mice surviving metastasis free throughout the experiments up to 160 days after PCa cell injection. These results agree with a model where T-DM1 eliminates quiescent PCa single cells or cell clusters soon after dissemination to prevent or delay recurrence and metastasis development much later.

Next, we examined a potential mechanism by which T-DM1 may lead to the increased survival in the *in vivo* model. Because T-DM1 is believed to kill cells in part through the induction of antibody-dependent cellular cytotoxicity (ADCC) [27,28] we tested whether T-DM1 induces ADCC on PC3 cells using splenocytes collected from severe combined immunodeficient (SCID) mice. Although SCID mice are able to accept xenografts, they retain production of natural killer cells, the cells that are responsible for ADCC [29]. Therefore, part of the activity of T-DM1 in the *in vivo* experiment may have resulted from immune cell activation. PC3 cells were labeled with the fluorescent dye, DiD, for subsequent identification and cultured in 1% FBS for 24 hours, and then incubated with T-DM1 and splenocytes. After 18 hours, PC3 cells were identified as DiD-positive cells, and dead cells were detected as DAPI positive (Fig. 6D). Our results showed that T-DM1 kills ∼ 20 % of the target cells by ADCC (Fig. 6E). Therefore, part or all of the activity of T-DM1 in our *in vivo* model could have been immune dependent despite the use of SCID mice.

## Discussion

Quiescent PCa cells are a common component in tumors but difficult to identify and study. Therefore, we used fluorescent reporters for cell cycle phases to separate PC3 PCa cells into G_1_ and G_0_ populations and performed proteomics analysis of purified membrane proteins to identify new potential cell surface markers for both research and treatment approaches. Gene ontology and pathways of the differentially expressed genes identified several potential gene groups, including nucleocytoplasmic transport. However, in part because purified membrane proteins also include intracellular membrane constituents, this approach did not identify an obvious candidate protein on the cell surface. Therefore, we manually examined the proteomics data and noted that membrane bound HER2 was more abundant in G_0_ than G_1_. HER2 was of particular interest to us because of the many available drugs that could be used to target it, and therefore we decided to explore HER2 further.

To validate the proteomics findings, we found that PCa cell surface HER2 was increased when quiescence was induced by serum starvation, the CDK inhibitor abemaciclib, co-culture with pre-osteoblasts, and injection into mice. Cell surface HER2 also correlated with nuclear endogenous p27. Therefore, cell surface levels of HER2 seem to increase in quiescent PCa cells under many different contexts of quiescence induction. For these reasons, we explored HER2 targeting as a potential therapeutic approach. Four treatments with the HER2 targeted antibody drug conjugate T-DM1 up until 12 days after intracardiac injection of PC3 cells into SCID mice delayed and prevented formation of metastases weeks and months later. This suggest that HER2 is deserving of more attention as a PCa therapeutic target, especially for use as adjuvant therapy or in the minimal residual disease setting. Our model of metastasis free survival after intracardiac injection best mimics systemic adjuvant therapy – in which patients at high risk of relapse after curative intent surgery or radiation to a primary prostate tumor are given medicines to prevent recurrence. However, clinical trials in this setting are notoriously difficult to conduct because of the extremely long time required for meaningful results. However, as at least some quiescent cells are present almost all prostate cancer patient specimens, other settings within the PCa clinical disease course are likely to be more practical to explore first.

Despite the robust increase in cell surface HER2, and potential as a quiescent PCa cell therapeutic, we do not think that HER2 will be useful as a research tool to identify quiescent PCa cells, at least on its own. While cell surface HER2 is increased in conditions where quiescence is induced, it does not effectively divide the population into two or more subpopulations, but rather results in a shift of the curve – for example see Fig. 2B. Therefore, at least with a flow cytometry approach, there is no objective way to divide the cell population into G_0_ and non-G_0_ fractions using HER2 levels alone. A combination of HER2 with other markers may eventually prove useful in this regard. Our finding that HER2 levels increase on the cell surface of PCa cells under quiescence-inducing conditions is unexpected because HER2 is involved in growth factor pro-proliferative signaling, and is often correlated with high levels of proliferation and HER2 signaling is upregulated in cycling cells. Although we do not yet know the mechanism, we postulate that high HER2 on the cell surface of quiescent cells may be due to reduced HER2 internalization and possibly correlate with lower HER2 pro-proliferative signaling. Decades ago, EGF family receptors were found to be decreased on the cell surface in response to serum deprivation [30]. However, our data are not consistent with this being the only mechanism at play because surface HER2 also correlates with proliferative state of cells within a population – not just as induced by serum deprivation or other perturbations. Perhaps, increased HER2 on the cell surface of a subset of quiescent cells may play a role in poising G_0_ cells to re-enter the cell cycle.

Therefore, we view our findings predominantly from the perspective of their potential impact on PCa biology and treatment. After the initial successes of HER2 targeted treatments in breast cancer, several clinical trials were performed in PCa, largely with disappointing results [31]. Because, unlike breast cancer, the HER2 (*ERBB2*) gene is rarely amplified in PCa, investigators have proposed that effective HER2 based therapies in PCa will depend on patient selection and / or combination therapies with agents targeting other EGF receptor family molecules or additional molecular targets [31]. Minner et al found only 0.04% of PCa patients has HER2 gene amplification, though they did find that high levels of HER2 expression did correlate with high proliferative rate and poor prognostic factors [32]. Despite the initial disappointing clinical results, laboratory investigations have continued to HER2 as an important protein in PCa biology. Several publications have pointed to the importance of HER2 and other EGF family receptors for PCa stem like cells and tumorigenesis – of special note to the present study, as we and others have identified overlap between PCa quiescence and stemness [13]. Day et al found that EGFR was particularly important for initial PCa tumor initiation and that EGFR (ERBB1) was expressed on many PCa patient circulating tumor cells. They also found that HER2 (ERBB2) was enriched in experimental bone metastases and that dual inhibition of EGFR and HER2 was required for effective bone metastasis treatment [20]. On a related note, Maillet et al fround that HER2 expression by circulating tumor cells in patients with metastatic castration resistant PCa correlated with worse clinical outcome, most importantly overall survival [33]. In testing response of PCa cell lines to trastuzumab (anti-HER2) and cetuximab (anti-EGFR), Andersson et al did not observe additional effects of addition of cetuximab to trastuzumab. However, these antibodies do not inhibit kinase activity directly, and the studies were carried out *in vitro*, which precluded a contribution from immune mechanisms [34].

Importantly, given the impact of androgen receptor (AR) signaling in PCa, investigators have recently found that prominence of HER2 signaling correlates AR signaling as well. Han et al performed transcriptomic profiling of metastatic castration resistant PCa patient specimens and patient derived xenografts and proposed three main groupings; AR high, neuroendocrine / small cell, and “mesenchymal stem cell like” and subsequently found that HER2 and HER3 (ERBB3) were key drivers of the mesenchymal stem cell like group [35]. They also found that neuregulins, which can bind to other EGF receptor family members and form heterodimers with HER2, caused enzalutamide resistant LNCaP cells to enter the cell cycle [35] – a key finding in light of our data finding a role for HER2 in quiescenct cells. Similarly, Karthaus et al found that neuregulins derived from stroma were important for regeneration of the normal mouse prostate after castration. Therefore, HER2 may help both normal and malignant prostate cells overcome androgen deprivation.

Overall, our findings of increased surface HER2 in quiescent PCa cells, are intriguing in light of the importance of HER2 in PCa stem cell biology, response to androgen deprivation, and wealth of available therapeutics. We think there is much more to discover for a role of HER2 in quiescent PCa cancer cells both in the lab and hopefully leading to the clinic.

## Methods

*Cell culture*: The human prostate cancer cell lines, PC3 (Cat #: CRL-1435), DU145 (Cat #: HTB-81), LNCaP (Cat #: CRL-1740), and an osteoblast precursor cell line, MC3T3-E1 (Cat #: CRL-2593) were obtained from the American Type Culture Collection. The C4-2B is a derivative subline of human prostate cancer LNCaP, originally isolated from a lymph node of a prostate cancer patient [36]. Normal human prostate epithelial PNT2 cells (Cat #: 95012613) was purchased from Sigma (St. Louis, MO). The human breast cancer cell lines, SKBR3 and BT-474 were gifts from Dr. Max S. Wicha (Department of Internal Medicine, University of Michigan Comprehensive Cancer Center). Cell line identities were confirmed by short tandem repeat (STR) profiling. Luciferase-expressing prostate cancer cells were established by lentiviral transduction. All prostate cancer cell lines, normal human prostate epithelial PNT2 cells and BT-474 were routinely grown in RPMI 1640 (Life Technologies, Cat #: 11875-093) supplemented with 10% (v/v) fetal bovine serum (FBS) (GEMINI Bio-Products, Cat #: 900-208), 1% (v/v) penicillin-streptomycin (Life Technologies, Cat #: 15140-122). SKBR3 cells were grown in DMEM (Gibco, Cat #: 11960) containing 10% (v/v) FBS, 1% (v/v) penicillin-streptomycin, 1% (v/v) Glutamax (Gibco, Cat #: 35050-061) and 1 mM sodium pyruvate (Gibco, Cat #: 11360). MC3T3-E1 cells were grown in minimal essential medium (MEM) alpha (Life Technologies, Cat #: 12561-056) containing 10% (v/v) FBS, 1% (v/v) penicillin-streptomycin. All cells were maintained at 37°C, 5% CO2, and 100% humidity. For serum starvation experiments, cells (1 × 10^5^ cells) were seeded in 6-well plates in culture media containing 10 % (v/v) FBS. The following day, cells were rinsed with phosphate buffered saline (PBS) once and cells were continued to culture in culture media without FBS for 3 days. For analysis of cell cycle with BrdU, PC3 cells were grown in 1% FBS RPMI and pulsed with BrdU for one hour. The BrdU was from BD Biosciences (# 552598)

*PC3 integrated with cell cycle reporters*: To develop a method to identify dormant cells, we transduced a human prostate cancer cell line, PC-3 with lentiviruses containing the fluorescent ubiquitination-based cell cycle reporters [12,37]. Both of the CDT1-mCherry reporter (pRetroX-mCherry-hCdt1_(30-120),_ Takara) and the p27 cyclin-dependent kinase inhibitor protein -Venus reporter (pMXs-IP-mVenus-p27, kindly provided to T. Oki, RIKEN) were packaged into lentivirus at the University of Michigan Vector Core Facility. PC-3 cells infected with both of the lentiviral reporters were selected for 7 days in RPMI media containing 10 μg/ml puromycin. To isolate the cells which are successfully integrated with both reporters, mVenus and mCherry double positive cells were sorted by FACS. Isolated PC-3 Venus mCherry (PC3VC) cells were cultured in the RPMI containing 10% FBS. p27-Venus is upregulated upon entry into quiescence and is tagged for degradation by the Kip1 ubiquitination-promoting complex (KPC) in late G_1_ and the Skp2 ubiquitin ligase in the G1-S transition. Therefore, this reporter is high during G_0_, but low upon G_1_ entry and the G_1_-S transition [12]. The Cdt1-mCherry reporter is high during G_0_ and G_1_ but degraded during S phase by Skp2-dependent degradation [12]. Together, these two reporters can be used to identify dormant cells. Using this system, we isolated cells in G_0_ or in G_1_ phase by FACS.

*Extraction of membrane proteins*: PC3-VC cells were cultured in the presence of 5% fetal bovine serum (FBS) for 48 h, and cells were treated with Accutase (Innovatove cell technologies, Cat #: AT104-500) for 10 min at room temperature to harvest from culture flasks. The cell cycle reporter's expression was analyzed by FACS, and G0 (p27+ Cdt1+) or G1 cells (p27- Cdt1+) were sorted by FACS. Membrane proteins were extracted from isolated PC3 cells in G0 or G1 phase using MEM-PER Plus Membrane Protein Extraction kit (Thermo Fisher Scientific, Cat. # 89842) following the manufacturer's directions.

*Flow cytometry*: The flow cytometric analyses and fluorescence-activated cell sorting (FACS) were performed on a FACS Aria II three-laser flow cytometer (Becton Dickinson, Franklin Lakes, NJ) and data were analyzed with FACS DIVA software (Becton Dickinson). BD cytometer setup & tracking beads (BD Biosciences, Cat #: 642412) were used for the daily instrument standardization and validation. Sorting calibration was performed before each sort by drop-delay using Accudrop beads (BD Biosciences, Cat #: 345249). Sorting of cells was performed using a 70 µm nozzle at 70 psi in purity mode.

*Proteomic mass spectrometry analysis*: Extracted membrane proteins (400 mg) in each of G0 and G1 samples were analyzed by proteomic mass spectrometry (Proteomics & Peptide Synthesis Core, University of Michigan) in order to identify and quantify proteins expressed in the samples. The mass spectrometry proteomics data have been deposited to the ProteomeXchange Consortium via the PRIDE [38] partner repository with the dataset identifier PXD037378 and DOI 10.6019/PXD037378

*Pathways and Gene Ontology Analysis*: The proteomics data was analyzed using Advaita Bio's iPathwayGuide™ in the context of pathways obtained from the KEGG database,[39] gene ontologies from the Gene Ontology Consortium database (2021-Nov4),[40] and network of regulatory relations from BioGRID: Biological General Repository for Interaction Datasets [41]. The analysis revealed 354 differentially expressed (DE) genes out of 2,685 genes with measured expression. Thresholds were set by us to recognize genes as DE, these were a p-value of 0.1 and a log (base 10) fold change of at least 0.6. P-values were calculated using Fisher's exact test.

*Analysis of cell surface protein expression by FACS*: Cell surface protein expression was analyzed by a FACS Aria three-laser flow cytometer (Becton Dickinson, Franklin Lakes, NJ). Cells were treated with Accutase (Innovatove cell technologies, Cat #: AT104-500) for 10 min at room temperature to harvest from plates. Cells were stained with fluorescence conjugated primary antibodies in PBS containing 1% FBS for 30 min at 4 °C. The following antibodies were used: HER2 (130-106-697), Mouse IgG1K isotype (130-113-761) obtained from Miltenyi Biotec. For cells also labeled with an intracellular antigen, the cells were labeled with Her2 prior to fixation and permeabilization. For p27 labeling,Cells were fixed with cold 70% ethanol and stained with anti-p27 antibody (R&D systems, Cat #: IC2256V) in PBS containing 1% FBS for 30 min at room temperature. Monoclonal mouse IgG2B (R&D systems, Cat #: IC0041V) was used as isotype control. For detection of DNA synthesis, an APC-conjugated antibody and associated buffers was from BD Biosciences (# 552598).

*Immunofluorescence*: Cells were fixed with 100% methanol (ice-cold) for 15 min at -20 °C. After the removal of methanol, cells were rinsed three times in PBS for 5 min each. To detect HER2 and p27, anti-HER2 (1:200) (Cell signaling, Cat #: 2165), anti-p27 (1:1600) (Cell signaling, Cat #: 3698) were used as the primary antibodies, respectively. Anti-rabbit IgG conjugated with Alexa Fluor 594 (Cell Signaling Tech, Cat #: 8889) and anti-mouse IgG conjugated with Alexa Fluor 488 (Cell Signaling Tech, Cat #: 4408) were used as the secondary antibodies. The slides were mounted with ProLong Gold antifade reagent with DAPI (Invitrogen, Cat #: P36931). Images were taken with a Nikon C1 confocal microscope.

*Co-culture experiments using PCa cells and OBs in vitro:* MC3T3-E1 cells (4 × 10^5^ cells) were seeded in 6-well plates in MEM-alpha containing 10 % (v/v) FBS, and 24 h later, PCa cells (4 × 10^4^ cells) were plated on the subconfluent MC3T3-E1 cells and cultured for 3 days.

*Western Blotting*: Cell lines were plated in 6 well plates at a density of 1.5 × 10^5^ cells per well in 10% FBS medium for 2 days, then grown in 10% FBS vs. serum free medium for 2 days. Cells were lysed with RIPA buffer supplemented with Halt protease inhibitor and phosphatase inhibitor cocktail. Samples were clarified and protein levels quantified via Pierce BCA Protein Assay. They were then mixed with 4x Lemmli SDS reducing sample buffer and separated on 4-15% Mini-Protean TGX Stain Free Protein Gels (Bio-Rad, 4568085) using Tris/Glycine/SDS running buffer, then transferred to PVDF membranes. Membranes were blocked in EveryBlot Blocking Buffer (Bio-Rad, 12010020), then probed with Her2/ErbB2 XP Rabbit mAb diluted 1:1000 (Cell Signaling Technology #4290S) and anti-Rabbit IgG1 - HRP- linked secondary Ab diluted 1:3000 (Cell Signaling Technology #7074S). Blots were visualized using SuperSignal West Pico PLUS Chemiluminscent Substrate (Thermo Fisher, 34580) on the Bio-Rad ChemiDoc Touch Imaging System. For loading controls, blots were stripped with with Restore™ Western Blot Stripping Buffer (Thermo Scientific #21059) and reprobed for β-actin (Cell Signalling Technology #4970L).

*HER2 expression on the surface of DTCs in vivo:* PC3 cells (1 × 10^6^ cells) were injected into male CB.17. SCID mice (8-10 weeks of age: Charles River, Wilmington, MA) by intracardiac left ventricular (i.c.) injection. Pelvis, femora and tibiae were harvested 48 hours after the injection, and the bones were crushed with a mortar and pestle and strained to remove debris. All steps used cold PBS buffer with 1% FBS unless otherwise noted. Single cell preparations were incubated with a FITC- anti-HLA-ABC antibody (BioLegend, Cat #: 311404) (dilution; 1:11), PE-Cy7-anti-mouse MHC (H-2Db) antibody (BioLegend, Cat #: 111516) (1:40), and APC-anti human HER2 antibody (Miltenyi Biotech, Cat #: 130-106-696) (1:11) for 30 min at 4 °C. After washing cells with PBS buffer with 1% FBS, dead cells were excluded by DAPI staining. Thereafter, DTCs of PC3 were identified as HLA-ABC positive, and mouse-MHC negative cells and the cell surface expression of HER2 on the DTCs was elucidated.

*A xenograft model of prostate cancer metastasis*: GFP/luciferase-expressing PC3 cells (5 × 10^5^ cells) were suspended in 100 μl of PBS and injected into male CB.17. SCID mice “Fox-Chase SCID” (10–12 weeks of age: Charles River Labs strain code 236) by left ventricle i.c. injection. For analysis of metastasis free survival, bioluminescence images were acquired after injection of luciferin using a PerkinElmer IVIS 2000 system. Animals that had a large portion of the signal in the lungs (indicative of a right ventricle injection) were removed from the analysis *a priori*. Time to metastasis formation visible by bioluminescence was then determined from the images. The data were analyzed by Kaplan-Meier analysis and the log-rank test. All experimental procedures were approved by the University of Michigan Committee for the Use and Care of Animals.

*Cell viability assays*: PC3 cells (1,000 cells) were plated into 96-well plates in growth medium with 10 % or 0.5% FBS. The following day, cells were treated with purified human IgG (1 or 10 mg/ml) (MP Biomedicals, Cat #:855908), T-DM1 (1 or 10 mg/ml) or Docetaxel (1 mg/ml) and viable cell numbers were evaluated by Cell Counting Kit-8 (CCK-8) (Dojindo, Cat #:CK04-13). Optical intensities were read on a multiwell scanning spectrophotometer at OD 450 nm (Thermo Labsystems). T-DM1 and Docetaxel were purchased from University of Michigan Cancer Center Pharmacy.

*Flowcytometric ADCC analysis*: ADCC analysis was performed by flow cytometry as previously described [42]. Briefly, we used PC3 cells as the target cells and mouse splenocytes collected from SCID mice as the effector cells. DiD-labeled PC3 cells were cultured overnight in a 96-well culture plate at 37 °C. Splenocytes were cultured in DMEM containing 10% FBS and IL-2 (Miltenyi Biotech, Cat #: 130-097-742) (1 ng/ml) for 24 h before ADCC assays. PC3 cells were treated with T-DM1 (10 mg/mL) for 30 min and splenocytes were added to the culture (5:1as the effector: target (E:T) ratio). The culture media was DMEM containing 1% FBS and IL-2 (1 ng/ml). The plate was further incubated for 18 h at 37 ˚C (5% CO2, humidified atmosphere). The cells were harvested and stained with DAPI (0.5 µg/ml) which stains dead cells. After 20 min of incubation, cells were subjected to FACS analysis.

*Cell labeling with DiD dye*: PC3 cells were stained with DiD dye (Molecular Probes, Cat #: v22887), according to manufacturer directions. Briefly, cells (1 × 10^6^ cells / ml) were incubated with DiD dye (0.5 μM) in serum-free conditions at 37 °C for 20 min, and then were washed three times with serum-free medium.

*Statistical Methods*: All numerical data were expressed as mean ± standard deviation (S.D.) unless specified otherwise. Two-tailed, unpaired Student's *t*-test was used for data analysis, with p < 0.05 considered to be statistically significant. Metastasis free survival was analyzed by Kaplan Meier plots and the log-rank test performed with GraphPad Prism software. Fisher's Exact Test performed with GraphPad Prism was used for read counts of proteomics data.

## Supplemenatary material

Table 1. Plasma membrane proteins with increased expression in G_0_. Forty proteins from the proteomics data with predicted plasma membrane localization, at least a four-fold higher expression in G_0_ phase compared to G_1_, and *p* < 0.1 by Fisher's exact test.

Table 2. Plasma membrane proteins with increased expression in G_1_. Twenty-eight proteins from the proteomics data with predicted plasma membrane localization, at least a four-fold higher expression in G_1_ phase compared to G_0_, and *p* < 0.1 by Fisher's exact test.

## CRediT authorship contribution statement

**Kenji Yumoto:** Investigation, Methodology, Validation, Data curation, Writing – original draft, Visualization. **Jibraan Rashid:** Formal analysis, Data curation, Writing – original draft, Writing – review & editing, Visualization. **Kristina G. Ibrahim:** Methodology, Investigation. **Steven P. Zielske:** Investigation, Visualization. **Yu Wang:** Validation, Investigation. **Maiko Omi:** Methodology, Investigation. **Ann M. Decker:** Investigation, Writing – review & editing. **Younghun Jung:** Investigation, Writing – review & editing. **Dan Sun:** Methodology, Investigation. **Henriette A. Remmer:** Methodology, Investigation, Formal analysis, Data curation. **Yuji Mishina:** Methodology, Writing – review & editing. **Laura A. Buttitta:** Conceptualization, Methodology, Resources, Writing – review & editing, Supervision, Funding acquisition. **Russell S. Taichman:** Conceptualization, Writing – original draft, Writing – review & editing, Supervision, Project administration, Funding acquisition. **Frank C. Cackowski:** Conceptualization, Methodology, Investigation, Data curation, Writing – original draft, Writing – review & editing, Visualization, Supervision, Project administration.

## Declaration of Competing Interest

The authors declare that they have no known competing financial interests or personal relationships that could have appeared to influence the work reported in this paper.

## Appendix Supplementary materials

Image, image 1

Image, application 2
