# Supplementary figures and images for: HER2 as a potential therapeutic target on quiescent prostate cancer cells
Source: Transl Oncol. 2023 Feb 18;31:101642. doi: 10.1016/j.tranon.2023.101642 (PMC9971552; doi:10.1016/j.tranon.2023.101642)

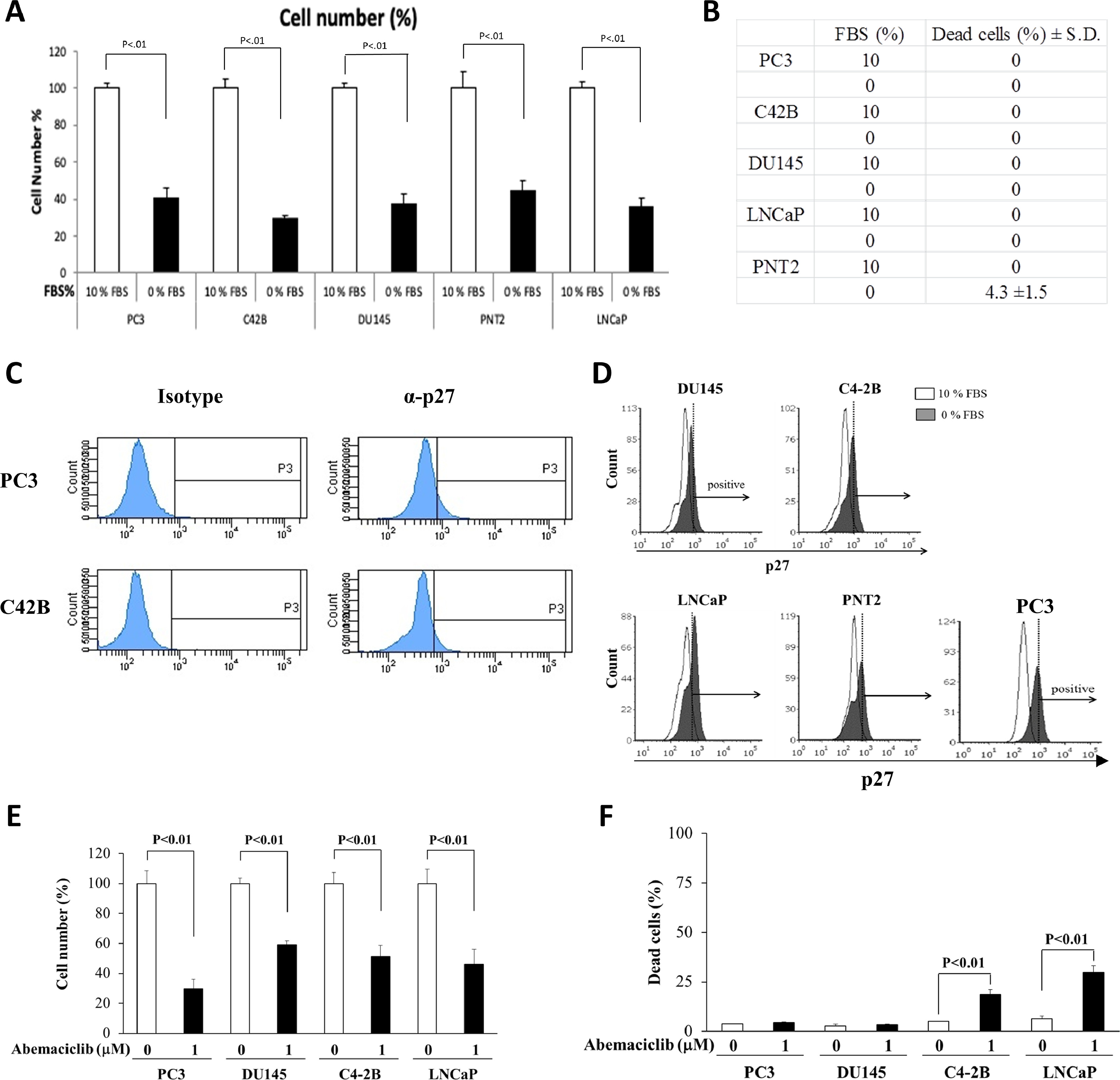

Supplement: Supplementary file 1 [file mmc1.jpg]
